# Quality of life in China rural-to-urban female migrant factory workers: a before-and-after study

**DOI:** 10.1186/1477-7525-11-123

**Published:** 2013-07-23

**Authors:** Chunyan Zhu, Qingshan Geng, Hongling Yang, Li Chen, Xianhua Fu, Wei Jiang

**Affiliations:** 1 Department of Preventive Medicine, School of Public Health, Guangzhou Medical University, Guangzhou 510182, P.R. China; 2 Department of Health of Guangdong Province, South Road, Guangzhou City martyrs on the 17th Provincial Health Office of the compound buildings, Guangzhou 510060, P.R. China; 3 Guangzhou Women and Children’s Medical Center, Guangzhou 510623, P.R. China; 4 Department of Internal Medicine, Department of Psychiatry & Behavioral Sciences, Duke University Medical Center, Durham, NC, USA

**Keywords:** Health-related quality of life, Job satisfaction, Female, Migrants, Before-and-after study, China

## Abstract

**Background:**

Rural-to-urban female migrant workers have a lower quality of life compared to the general population. Improving these conditions remains highly challenging. This paper reports the health-related quality of life (HRQoL) of the female migrant workers in an educational project.

**Methods:**

In this before-and-after study, a community-based health education intervention was developed to improve female migrant workers’ HRQoL and job satisfaction. A factory was selected as the location to implement the trial, using a before-and-after design. The education intervention included distribution and free access to study materials, monthly lectures, and counseling. The primary endpoint was HRQoL, and gynecological disease and job satisfaction were secondary endpoints. We assessed HRQoL at baseline and at 6-month follow-up using the Health Survey Short Form (SF-36).

**Results:**

Compared to the baseline assessment, the participants at the 6-month survey reported higher General Health scores (standardized-*β* coefficients (Betas) of *β* = 0.056; *P* <0.001), Vitality scores (*β* = 0.066; *P* <0.001), Mental Health scores (*β* = 0.062; *P* <0.001), mental component summary scores (*β* = 0.040; *P* <0.001), and job satisfaction (Odds Ratio [OR] 2.104, 95% confidence interval [CI] 1.837-2.408; *P* <0.01).

**Conclusions:**

A community-based educational intervention, targeted at female migrant workers, appears effective in improving HRQoL and job satisfaction.

## Background

With the rapid economic development in China, the population move from rural areas to urban areas is increasing, and at least 50% of this population is young females [1,2]. Rural-to-urban migrant workers are a special majority group in Shenzhen city of China, and most of them range from 16 to 25 years old. The migrant workers are usually of lower social status, have a heavy physical work load, high quantitative demands, long working hours (10–12 h per day), lower income, insufficient protection from social security agencies and poor labor protection [3]. The health-related issues and health-related quality of life (HRQoL) in the rural-to-urban female migrants are recognized increasingly and cause for concern [4]. One previous study had explored the HRQoL and related factors of rural-to-urban female migrant workers living in the factories in Shenzhen city of China with a cross sectional study, and its results showed that the subjects suffered from poor health and demonstrated considerable decrements (compared with general rural women) on seven SF-36 domains (except for physical functioning) [5].

Quality of life (QOL) is defined by WHO as “an individual’s perception of their position in life in the context of the culture and value systems in which they live, and in relation to their goals, expectations, standards and concerns” [6]. Health-related QOL (HRQoL) is one of the essential aspects of human health, which is embedded in a physical, mental and social context. Over the past few decades, measuring HRQoL has become a common approach in health research. A wide variety of assessment instruments are now available, and it has increasingly received attention in the context of monitoring various health aspects [7].

The decrements on SF-36 domains are attributable to preventable and behaviorally modifiable causes. Previous studies have demonstrated that HRQoL can be influenced by multiple factors, such as age, educational level, physical activity, occupational stress, working hours, occupation and disease [8,9]. The socioeconomic status factors such as education, income, occupational status, or access to health insurance are hard to change, while lifestyle and behavioral factors such as sports activity, diet, sleep, reducing stress, and improving relationships, are easier to change. Poor QOL has been found to be strongly associated with reduced work performance and early retirement [10]. While the extent to which a preventive package of evidence-based interventions at the community level could improve HRQoL is unknown. Estimates based on clinical data suggest that a program of lifestyle weight management adapted for African-Americans that produced modest weight loss during the intensive phase of the study was associated with short-term improvements in general health and vitality domains [11]. However, few studies were conducted in the rural-to-urban female migrant workers to examine the impact of health education on HRQoL [12].

Studies showed that health promotion activities in the workplace had the capacity to influence both individuals and populations [13,14]. The workplace can be considered a microcosm of larger society and as such can provide an effective setting for addressing both individual health and the health of populations. Programs in the workplace can reach and engage segments of the population that may not otherwise be exposed to health-improvement efforts [15]. The results of a quasi-experimental study carried out among staff working with intellectual disabilities people showed a significant reduction in stress and exhaustion, and a strong significant rise in job satisfaction after intervention [16]. To test the efficiency of an intervention, other researches applied didactic and process-oriented strategies to improve job satisfaction and quality of life in Palliative care (PC) nurses. Its results showed that Palliative care (PC) nurses in the experimental group reported more perceived benefits of working in PC after the intervention and at follow-up; however, spiritual and emotional quality of life remained unaffected by the intervention [17].

Identification of an effective approach to preventive care that builds on existing capacities and accelerates program effectiveness is important [18]. The limited success of large-scale studies on behavior change interventions has been attributed to poor consideration of the social context that shapes behaviors, while treating individual health behaviors as stand-alone entities [19,20].

Therefore, the aim of our study was to determine the effectiveness of a community based, socio-culturally contextualized, lifestyle and behavioral educational program on Chinese female migrant factory workers’ HRQoL and job satisfaction. To our knowledge, this is the first community-based reproductive health, mental health and occupational health education intervention study for rural-to-urban female migrant workers in China.

## Methods

### Study design

This study used a before-and-after design. A community-based health education intervention was developed to improve rural-to-urban female migrant manual workers’ HRQoL, health and job satisfaction, and to promote their health literacy. Two cross-sectional surveys were carried out before and after the intervention. This study was conducted between July-August, 2010 and March-April, 2011. The trial was approved by the Ethics Review Committees of Guangzhou Medical University (Guangzhou, China). All participants provided voluntary informed consent.

### Study site and population

An overseas-funded factory of Bao’an District in Shenzhen city was selected for this study. Details of the study location have been previously published [5]. The Health Bureau of Bao’an District helped to locate the factory. The inclusion criteria of the factory were: (1) Overseas-funded manufacturing factory that had almost zero social interaction with outside; (2) The factory would have 20,000 or greater number of workers and at least 80% of them would be rural-to-urban female migrant workers; (3) The managers of the factory would be willing to cooperate with the implementation of the study; (4) Community-based health services provided by the local government were in place in the factory for these workers. Rural-to-urban female migrant manual workers in the factory were the study population. Because of far from the town, strict management system and the 8-hour shifts (10–12 h of actual work per day) of the factory, the workers rarely communicated with outside. The factory has one community health center on the main campus, with at least five doctors who provide the usual community-based health service and occupational health services.

### Sample size

Reproductive health and sexually transmitted diseases (STD) account for a high proportion of health problems among female migrant workers in China, and these diseases were associated with reduced HRQoL. The results in this current paper are part of a community trial, which was to advance the reproductive health, HRQoL and job satisfaction of female migrant workers. Sample size was calculated to provide estimates of the prevalence of reproductive health problems among female workers for each cross-sectional survey. Based on the demographic characteristics of the factory and the annual visits to the community health-care center in the factory, we estimated a prevalence of reproductive health problems among female workers at approximate 30.0%. With 80% power at 5% significance level, a minimum of 934 participants was required for each survey.

### Intervention

The intervention was designed to promote the HRQoL, health and job satisfaction of female migrant factory workers, which was designed according to the sociocultural context of the female factory workers (female, young, lower education level, lower social status, heavy physical work load, long working hours, lower income, and high prevalence of reproductive and sexual ill-health). During the intervention period, the female migrant workers in the factory received both the usual community-based health services provided by the local government and the intervention package. The intervention package consisted of free access to educational materials and lectures about reproductive health, mental health and occupational health that included the lifestyle and behavioral intervention (especially, physical activity, dietary habits, personal hygiene, sexual behaviors and occupational protection), coping strategies and stress management, video educational materials and propaganda column. Those learning materials were broadly distributed to female workers in the factory (Table 1). Prior to the actual implementation of the educational intervention, the study investigators collected comments from the community health care providers, representatives of workers and administrators of the factory for refining the study procedures and ascertaining a successful implementation.

**Table 1:** Components of the intervention

	***Process and coverage***
Training the factory clinicians	Medical experts in reproductive health, mental health and occupational health provided training to the clinicians aiming at providing related health knowledge to the female workers.
Lectures given by experts	1. The same experts provided the training to clinicians delivered free monthly lectures which were about the knowledge, skills, information session on available preventive services of reproductive health, mental health and occupational health.
	2. Each lecture lasted for 2 hours;
	3. Six lectures were carried out and approximate 750 female workers participated.
Distribution of educational material hand-out	1. Reproductive health, mental health and occupational health education materials are organized in a booklet, respectively and distributed in various places, such as to factory workshops, to newly recruited workers who received job trainings in the training center, monthly to the dormitories where female workers resided, and free provision of educational materials in the community health center.
	2. Approximate 20,000 booklets were distributed.
Video educational materials	1. Video educational materials were broadcasted daily from 9 AM to 10 AM and from 7 PM to 8 PM in the community health center;
	2. During the intervention, the broadcast of the Video educational materials were repeated three times every day to ascertain coverage among all workers across shifts.
Propaganda column	1. Eight displays of the study educational information were established in the community center and the workshops which were renewed monthly;
	2. The contents of the display complemented to the lectures.

### Data collection

HRQoL was assessed using the Health Survey Short Form (SF-36), which was administered at the baseline visit and at the 6-month follow up. The SF-36 is a 36-item, generic quality of life measure. It contains four domains of physical health and four of mental health. The pormer includes Physical Functioning (PF), Role-Physical (RP), Bodily Pain (BP), General Health (GH); the latter contains Vitality (VT), Social Functioning (SF), Role-Emotional (RE), and Mental Health (MH) [21,22]. Each domain’s score is on a scale from 0 (worst) to 100 (best), the higher scores the better the function of individual. These eight domains are weighted and summed to calculate the physical component summary (PCS) and the mental component summary (MCS) scores, which represent subjective physical health and mental health, respectively. PCS and MCS were rated according to the SF-36 users’ manual [23,24]. The SF-36 has been validated extensively and has been established as the best generic measure of QOL among general and diseased populations due to its sound psychometric properties, brevity and comprehensiveness [22,25].

Gynecological diseases were considered based on the self-reported presence of illnesses via questions like “Has a doctor or other health care provider EVER told you that you had a gynecologic disease, such as menstrual disorder over the past six months?”, which were then confirmed by a medical doctor. Job satisfaction was assessed by self-report in response to the question “Are you satisfied with your current job?” inviting a response of “very dissatisfied”, “dissatisfied”, “no feelings either way”, “satisfied” or “very satisfied”.

Sociodemographic data included age, race/ethnicity, marital status, education level, work duration and occupation. Marital status was grouped as married, single and divorced. Education level was grouped as primary, secondary and university. Occupation was grouped as manual and non- manual. The age of the subjects ranged from 16 to 48 years, and we used dummy variables for age defined by <20 years old, 20 ~ 24 years old, 25 ~ 29 years old, and ≥30 years old.

Two cross-sectional surveys were conducted to obtain the research data during July-August, 2010 prior to the implementation of the education and six months later (March-April, 2011), using the same questionnaire, scale and method. Standard procedures were established to guide the recruitment, training, and supervision of these data collectors who received training prior to each survey to improve the skills and to reduce inter/intra variation of the interview/data collection. Data’s collectors and their supervisors were female students who were receiving undergraduate or master’s degree education in Guangzhou Medical University and were blinded from the baseline and the 6-month follow up, and independent of the education implementation. Information on demographic, HRQoL, gynecological disease and job satisfaction was collected via a face-to-face anonymous interview by those data collectors from randomly selected samples of all migrant female manual workers.

The representative sampling was primarily based on the distribution of the factory dormitories, which in both factories are grouped into five areas, east, west, and north, south and central. Randomly, two dormitory buildings were first selected from each of the five compounds of the factory, then two floors from each dormitory building, generating a sample of 20 floors in the factory for each data collection period. Although the size and the number of beds of each dormitory were identical and all dormitories were accessible to the study, the number of female workers in each varied at times. The final sample size was therefore different for each cross-sectional survey.

The primary outcomes were changes in HRQoL at baseline and the end of the 6-month intervention period, and Gynecological disease and job satisfaction were secondary endpoints.

### Statistical analysis

Student t-tests, Chi square or Wilcoxon Tests were used to test the differences between the baseline and the 6-month assessments in respect of continuous and categorical measures. Adjusted for sociodemographic variables, job satisfaction and Gynecological disease, multiple linear regression models were used to identify effects of the intervention on the SF-36 domain scores and summary scores (PCS/MCS). And multivariate regression models were used to identify effects of the intervention on job satisfaction and Gynecological disease by adjusted for sociodemographic variables. Intervention period was over the 6-month intervention period and the data were from the baseline and the 6-month follow up.

The intervention effect was estimated using the Standardized Coefficients of the SF-36 domain scores and summary scores (PCS/MCS), and the adjusted odds ratio (OR) of job satisfaction and Gynecological disease for the 6-month follow up relative to the baseline.

All data forms underwent scrutiny for logical inconsistencies, skip patterns and missing values. The data were coded and double-entered into a relational database on EpiData 3.1. All analyses were done using SPSS 15.0. *P* value less than 0.05 was considered statistically significant.

## Results

### Socio-demographic

The baseline survey recruited 3,344 female workers in the factory, and the 6-month survey recruited 1,740. The sample sizes exceeded the estimated sample sizes.

Table 2 demonstrated basic demographic characteristics of the study population. All Participants were female manual workers, and over 84.0% of them were single. The age of the study population ranged from 16 to 48 years. Most of them (>82.0%) were less than 25 years old. Among them, 74.0% received only elementary education and 70.0% participants were in the first year of employment in the participating factories.

**Table 2:** Key sociodemographic characteristics of participants at the baseline survey and at the 6-month follow up survey

		***Baseline (N1 = 3344,%)***	***6-month follow up (N2 = 1740,%)***	***P***
Age(year)	Mean ± SD	20.9 ± 3.4	21.9 ± 3.9	
	Min ~ Max	16 ~ 48	16 ~ 47	
Age(year)*	<20	1294(38.7)	727(41.8)	<0.001
	20 ~ 24	1691(50.6)	700(40.2)	
	25 ~ 29	250(7.5)	224(12.9)	
	≥30	109(3.3)	89(5.1)	
Education level	Primary	2473(74.0)	1294(74.4)	0.756
	Secondary	851(25.4)	421(24.2)	
	University	20(0.6)	25(1.4)	
Marital status	Single	2899(86.7)	1473(84.7)	0.047
	Married/Divorced	445(13.3)	267(15.3)	
Work duration (month)	<6	1712(51.2)	759(43.6)	0.245
	7~	646(19.3)	549(31.6)	
	≥13	986(29.5)	432(24.8)	

* 1 person was missing in the intervention group.

### Quality of life, gynecological disease and job satisfaction

Table 3 shows mean scores for HRQoL at baseline and at the 6-month follow up survey. The participants reported higher General Health scores, Vitality scores, Social Functioning scores, Mental Health scores and MCS scores (all *P*s <0.05) at the 6-month survey. And it was similar to those at the baseline survey on Physical Functioning scores, bodily pain scores, Role-Emotional scores and PCS scores (all *P*s >0.05). However, Role Physical scores were significantly lower relative to the baseline assessment (*P* <0.01). The participants at the 6-month survey were similar to those at the baseline survey on gynecological disease, and reported higher job satisfaction (25.8% vs. 13.4%; *P* <0.001) (Table 4). The study population at both baseline and 6-month follow-up survey were similar to those only at the 6-month follow-up survey on seven concepts’ scores of SF-36 (except for General Health), MCS scores, PCS scores, gynecological disease and job satisfaction (Ps >0.05) (Table 3 and Table 4).

**Table 3:** Pearson correlation coefficients between gynecological disease, job satisfaction and other socio-demographic parameters (N = 5084)

	**Gynecological disease**	**Job satisfaction**	**Age**	**Work duration**	**Education level**	**Marital status**
Gynecological disease	1.000	0.001	0.254	0.043	−0.032	0.300
		0.931	<0.0001	0.002	0.024	<0.0001
Job satisfaction	0.001	1.000	0.069	−0.045	−0.087	0.083
	0.931		<0.0001	0.002	<0.0001	<0.0001
Age	0.254	0.069	1.000	0.144	−0.076	0.633
	<0.0001	<0.0001		<0.0001	<0.0001	<0.0001
Work duration	0.0430	−0.045	0.144	1	−0.113	−0.006
	0.002	0.002	<0.0001		<0.0001	0.678
Education level	−0.032	−0.087	−0.076	−0.113	1.000	−0.088(**)
	0.024	<0.0001	<0.0001	<0.0001		<0.0001
Marital status	0.300	0.083	0.633	−0.006	−0.088	1.000
	<0.0001	<0.0001	<0.0001	0.678	<0.0001	

**Table 4:** Scores of each SF-36 domain and summary scores and multiple linear regressions for SF-36 domain scores and summary score PCS/MCS (Mean ± SD)

	***Baseline***	***6-month follow up***	***Baseline and 6-month follow up ***^***a***^	***Only 6-month follow up ***^***b***^	***Standardized coefficients (6-month follow up to baseline)***		***P***_***1***_^***d ***^***value***	***P***_***2***_^***e ***^***value***
					**Crude**	**Adjusted **^**c**^		
Physical Functioning (PF)	100.0 ± 0	100.0 ± 0	100.0 ± 0	100.0 ± 0	-	-	1.000	1.000
Role Physical (RP)	75.3 ± 31.5	72.4 ± 32.1	71.8 ± 31.6	72.5 ± 32.2	−4.027	−0.059**	0.002	0.775
Bodily Pain (BP)	79.5 ± 18.8	80.1 ± 19.4	79.6 ± 19.5	80.1 ± 19.3	0.344	0.008	0.295	0.712
General Health (GH)	65.3 ± 17.8	67.3 ± 18.5	64.5 ± 19.8	67.7 ± 18.3	2.178	0.056**	<0.001	0.021
Vitality (VT)	58.5 ± 17.6	62.8 ± 18.2	60.9 ± 17.6	63.0 ± 18.2	2.564	0.066**	<0.001	0.126
Social Functioning (SF)	83.1 ± 15.9	84.0 ± 15.3	84.4 ± 14.8	84.0 ± 15.4	0.523	0.015	0.040	0.720
Role-Emotional (RE)	68.8 ± 35.2	70.4 ± 35.2	66.7 ± 36.7	70.8 ± 34.9	−0.446	−0.006	0.133	0.118
Mental Health (MH)	70.3 ± 14.3	73.2 ± 14.4	72.0 ± 15.7	73.4 ± 14.2	1.938	0.062**	<0.001	0.192
PCS	53.5 ± 5.8	53.5 ± 6.1	53.0 ± 6.3	53.6 ± 6.1	−0.093	−0.007	0.751	0.242
MCS	46.8 ± 9.4	48.4 ± 9.6	47.4 ± 10.0	48.5 ± 9.5	0.818	0.040**	<0.001	0.115

Note: Physical Functioning was deleted from the analysis.

a: Study population participated both baseline and 6-month follow up survey.

b: Study population only participated 6-month follow up survey.

c: Adjusted for age, education level, marital status and work duration, job satisfaction and Gynecological disease.

d*:* Comparison of baseline and 6-month values.

*e:* Comparison of study population at both baseline and 6-month follow up survey and those only at 6-month follow up survey.

**: *P* < 0.01.

### Multivariate analyses

The Pearson’s correlation coefficients (*r*) among gynecological disease, job satisfaction and other socio-demographic parameters (age, work duration, education level and marital status) are presented in Table 3. Correlation analyses revealed weakly relationships among gynecological disease, job satisfaction and other socio-demographic parameters with the *r* values ranging from −0.113 to 0.633 (*P*s < 0.001). A moderate correlation was found between age and marital status (*r* = 0.633).

The results of multiple linear regression models of the QOL scores of each domain (except for physical functioning, of which the scores of all the objects were 100), the PCS scores and MCS scores, respectively, were shown in Table 4. After adjusting for age, education level, marital status, work duration, gynecological disease and job satisfaction, General Health scores (standardized-*β* coefficients (Betas) of *β* = 0.056; *P* <0.001), Vitality scores (*β* = 0.066; *P* <0.001), Mental Health scores (*β* = 0.062; *P* <0.001) and MCS scores (*β* = 0.040; *P* <0.001) were significantly higher, while Role Physical score (*β* = −0.059; *P* <0.001) was significantly lower at the 6-month follow-up assessment than at the baseline assessment.

The results of multivariate regression models for job satisfaction and Gynecological disease, respectively, were shown in Table 5. After adjusting for age, education level and marital status work, a pronounced improvement was noted in job satisfaction at the 6-month follow-up assessment than at the baseline assessment (OR 2.104, 95% CI 1.837-2.408; *P* <0.01) (Table 5).

**Table 5:** Gynecological disease and job satisfaction of participants at the baseline survey and at 6-month follow up survey

		***Baseline (N1 = 3344,%)***	***6-month follow up (N2 = 1740,%)***	***Baseline and 6-month follow up ***^***a ***^***(N3 = 194,%)***	***Only 6-month follow up ***^***b ***^***(N4 = 1546,%)***	***Odds ratio (6-month follow up to baseline)***		***P***_***1***_^***d ***^***value***	***P***_***2***_^***e ***^***value***
						**Crude**	**Adjusted**^***c ***^**(95% CI)**		
Gynecological disease #	Yes	379(11.3)	222(12.8)	166(85.6)	1352(87.5)	1.144	1.068(0.886, 1.288)	0.135	0.458
	No	2965(88.7)	1518(87.2)	28(14.4)	194(12.5)				
Job satisfaction##	Satisfied	448(13.4)	389(25.8)	15(8.2)	119(9.0)	2.088	2.104(1.837, 2.408)**	<0.001	0.464
	Neither satisfied nor dissatisfied	2397(71.7)	987(65.4)	127(69.4)	860(64.8)				
	Dissatisfied	499(14.9)	134(8.9)	41(22.4)	348(26.2)				
	Missing	0	230	11	219				

a: Study population who participated both baseline and 6-month follow up assessment.

b: Study population who only participated 6-month follow up.

c: Adjusted for age, education level, marital status and work duration.

d*:* Comparison of baseline and 6-month values.

*e:* Comparison of study population who participated both baseline and 6-month follow up assessment and study population who only participated 6-month follow up.

#: Binary Logistic regression model.

##: Ordinal regression model.

**: *P* < 0.01.

## Discussion

This study demonstrated that the implementation of the educational program that aimed at improving the HRQoL, health and job satisfaction for female migrant workers was associated with significant improvements in the SF-36 domain scores of General Health, Vitality, Mental Health and MCS, and job satisfaction. The educational interventions were developed based on findings from formative research [5] and the input of community health providers to address the fundamental needs of female migrant workers, and implemented with the active participation of the community administrative and health providers. The study highlights the importance of understanding the existing sociocultural context for translating scientific evidence into effective and sustainable health strategies at the community level.

The findings of the study are consistent with results of previous studies in countries outside of China. Research indicates that health promotion plays an important role in enhancing quality of life [26], and health education is enabled for a higher quality of life [27]. Lifestyle behavioral interventions have produced short-term improvements of HRQoL [28]. In a sample of obese African-Americans with modest weight change after a 10-week lifestyle intervention, the change in HRQoL was also modicums, and the Vitality domain of the SF-36 appeared to be the most responsive to small changes in weight for this study population [11]. Rana and colleagues reported that a community-based intervention covering three broad domains of health care management, health and social awareness related to elderly people’s health, and health care, increased the HRQoL in old age [29].

The results of our study also showed that a pronounced improvement was noted in job satisfaction at the 6-month follow-up assessment than at the baseline assessment (OR 2.104, 95% CI 1.837-2.408). The combination of high psychological work demands, low job control, and poor social support at work causes the greatest job strain [30]. Stressful work conditions may lead to low job satisfaction, physical and/or mental health problems in the workers [31]. Cimete and colleagues reported that a positive correlation was found between Quality of Life and job satisfaction among a sample of 501 nurses [32]. That is, people that reported greater Quality of Life were also more satisfied with their jobs. The relationship between job satisfaction and HRQoL, and the efficiency of the workplace -based educational interventions to improve quality of life and job satisfaction has still been little explored in China.

Among the influenced factors of HRQoL of the female migrant factory workers, the factors of age, educational level, lower social status, heavy physical work load, long labor hours, poor income, and insufficient protections from social security agencies are common and hard to change in China. While sports activity, eat a varied and nutritious diet, get enough sleep, don’t smoke, limit alcohol, reducing stress, and improving relationships, are easier to variety. Previous studies have shown that sports activity decrease occupational stress, is associated with a reduced risk of different diseases [33,34], and plays an important role in HRQoL [9]. More specifically, leisure-time physical activity was positively related to several HRQoL scales (Vitality, Mental Health, Bodily Pain, and Vitality), and MCS scores.

Coping resources included four aspects, i.e., recreation, self-care, social support and rational coping [35]. Recreation refers to the extent to which the individual makes use of and derives pleasure and relaxation from regular recreational activities. Social support refers to the qualitative aspect described as perceived social support, such as the content and availability of relationships with significant others. Both the COPE and the ways of coping sub-scales have been reliably tied to psychological distress, such that active coping strategies appear dependably to produce better emotional adjustment to chronically stressful events than do avoidant coping strategies. The previous epidemiological studies also supported evidence for the adverse effects of occupational stress on different health outcomes, such as psychiatric disorders [36] and self-reported health status [37]. Increasing coping resources may be a useful measure to relieve occupational stress, improve health outcomes, and leading to a better QOL [38].

The before-and-after studies measure provider performance before and after the introduction of an intervention (e.g. dissemination of guidelines) in the same study site(s) and any observed differences in performance are assumed to be due to the intervention [39]. The before-and-after design is relatively simple to conduct and offers better evidence about preliminary evidence for intervention effectiveness, especially when supplemented with complementary information than the other non-experimental observational designs. It is most useful in demonstrating the immediate impacts of short-term programs, and is less useful for evaluating longer term interventions. This is because over the course of a longer period of time, more circumstances can arise that may obscure the effects of an intervention [40]. These circumstances are collectively called threats to internal validity. Furthermore, in such studies, the intervention is confounded by the Hawthorne effect (the non-specific beneficial effect on performance of taking part in research) which could lead to an overestimate of the effectiveness of an intervention [41]. In order to minimize the threats to validity, the effective ways were carried out in this study. Firstly, an intervention diary was used to keep track of the significant events at the workplace throughout the intervention and evaluation, and none events affected the intervention outcome have occurred in the workplace during the study period. Secondly, to deal with most instrumentation threats, “before” and “after” measurement methods were constant and give questionnaires in the same format and conditions for all measurements.

At the same time, because of the high mobility of workers (more than 43% of workers were employed for less than six months in the study), most study population hadn’t received intervention for six months, thus the intervention effectiveness might have been minimized. Furthermore, the large mobility workers made a high rate of loss to follow-up, and 88.5% study population at the 6-month did not participate in the baseline assessment. Two cross-sectional surveys were done to obtain information at baseline and 6-month in the same factory. Thus conservative analytical methods recommended for analysis of randomized controlled trials have been used.

Despite these limitations, the study provides encouraging results suggest that a community (workplace)-based educational intervention program promoting health could lead to improvement in HRQoL and job satisfaction among migrant female workers in China.

## Conclusions

In conclusion, the results indicate that using before-and-after study to test the effectiveness of educational programs is feasible in certain communities in China, and implementation of educational intervention targeting on improving HRQoL among young migrant female workers is effective.

## Abbreviations

HRQoL: Health-related quality of life; PF: Physical functioning; RP: Role-physical; BP: Bodily pain; GH: General health; VT: Vitality; SF: Social functioning; RE: Role-emotional; MH: Mental health; PCS: Physical component summary; MCS: Mental component summary; OR: Odds ratio; CI: Confidence interval.

## Competing interests

The authors have no conflict of interest to disclose.

## Authors’ contributions

CZ directed the study implementation, including quality assurance and control, and analysis and interpretation of data, and drafting the article. QG designed the study and reviewed the article. HY designed the study’s analytic strategy and helped conduct the literature review. XF helped supervise the field activities and prepared the Discussion sections of the text. WJ helped design the study and revised the article critically for important intellectual content. All authors read and approved the final manuscript.
